# Protein Nutrition: Understanding Structure, Digestibility, and Bioavailability for Optimal Health

**DOI:** 10.3390/foods13111771

**Published:** 2024-06-05

**Authors:** Nneka Ajomiwe, Mike Boland, Suphat Phongthai, Manisha Bagiyal, Jaspreet Singh, Lovedeep Kaur

**Affiliations:** 1 School of Food Technology and Natural Sciences, Massey University, 4442 Palmerston North, New Zealand; 2 Riddet Institute, Massey University, 4442 Palmerston North, New Zealand; 3 Food Science and Technology Division, School of Agro-Industry, Chiang Mai University, Chiang Mai 50100, Thailand

**Keywords:** alternative proteins, plant proteins, protein digestibility, protein nutrition, protein structure

## Abstract

This review discusses different protein sources and their role in human nutrition, focusing on their structure, digestibility, and bioavailability. Plant-based proteins, such as those found in legumes, nuts, and seeds, may contain anti-nutritional factors that impact their bioavailability apart from structural and compositional differences from animal proteins. Animal proteins are generally highly digestible and nutritionally superior to plant proteins, with higher amino acid bioavailability. Alternative protein sources are also processed in different ways, which can alter their structure and nutritional value, which is also discussed.

## 1. Introduction

In any discussion of protein nutrition, it is important to understand the background of protein nutrition, its processes, and limiting factors. This brief review gives an overview and relevant background to this topic.

Proteins are essential macromolecules in biology, playing a crucial role in various physiological processes and contributing significantly to the nutritional quality of our diet. Their structure determines their digestibility, the bioavailability of amino acids, and the physiological responses they trigger in the body. The amino acid composition and bioavailability of dietary proteins are crucial, as they determine the nutritional quality of the protein source. Understanding protein structure and its connection to nutrition, digestibility, and bioavailability is necessary for enhancing dietary choices and ensuring the body receives adequate nutrients. Recent studies have highlighted the importance of a balanced intake of essential amino acids to support optimal growth, immune function, and overall health [1,2].

Depending on their source, dietary proteins have different levels of digestibility. Animal-based proteins obtained from meat, fish, cheese, and milk (dairy) are generally highly digestible, with a high proportion of their amino acids absorbed by the body. Plant-based proteins, on the other hand, often contain anti-nutritional factors and have complex structures that can reduce their digestibility. Cooking, processing, and preparation methods can influence protein digestibility. The structure of proteins can affect how they are broken down and digested in the gastrointestinal tract. The three-dimensional conformation of proteins can impact their susceptibility to enzymatic cleavage. Highly structured proteins may resist digestion, potentially limiting the availability of amino acids for absorption [3]. The structural makeup of proteins affects their nutritional value as well. Large fibrous protein complexes are more difficult to digest than smaller proteins, and some, such as keratin, are indigestible. Not all amino acids are absorbed and made accessible for utilisation by the body due to incomplete digestion of some fibrous proteins, which lowers their nutritional value [4]. Although plant-sourced proteins offer environmental and health benefits, and research increasingly includes them in study formulas, plant proteins have less of an anabolic effect than animal proteins due to their lower digestibility, lower essential amino acid content (especially leucine), and deficiency in other essential amino acids, such as sulfur amino acids or lysine [5]. Martínez and Larralde (1984), alongside other researchers, as cited in [5], conducted several studies and evaluated the effect of consuming plant-based proteins on muscle protein metabolism in young, adult, and old rats, pigs, and humans, compared to animal proteins, i.e., meat, milk, and its constitutive proteins (casein and whey proteins). The majority of these studies have reported that good-quality animal proteins have a greater ability to enhance the rate of muscle protein synthesis and support muscle mass than plant-based proteins. In general, animal proteins have a higher Digestible Indispensable Amino Acid Score (DIAAS) when compared to plant-based proteins, with some exceptions, such as soy protein and potato protein [6].

“Protein bioavailability” describes how well dietary proteins may be absorbed, used, and incorporated into the body’s metabolic functions. Factors affecting protein bioavailability include protein source, processing methods, and interactions with other dietary components. Recent investigations have investigated the bioavailability of specific proteins, including many plant-based proteins, and their potential to meet nutritional requirements [7]. The bioavailability of some proteins (rice, oat, corn, and soy protein) is very low due to their particularly dense structure and stable tertiary structure, leading to a large amount of protein resources wasted [8].

In addition to bioavailability, the rate of digestion can be important [9]. This is partly because rapid digestion leads to higher levels of amino acids in the bloodstream, which, in the case of branched-chain amino acids, can up-regulate protein synthesis, which is important for older adults, in the avoidance or treatment of sarcopenia, and also for athletes, who need to enhance muscle repair and build muscle mass [10]. It is also important because the microbiota of the large intestine can digest any protein that reaches it, thus making it unavailable to the host [11].

A further important consideration is gastric digestion, whereby some peptides leaving the stomach can be allergenic, reacting with immunogenic cells in the small intestine (Payen’s patches) or otherwise causing intolerance in susceptible individuals. This is further discussed below.

This review emphasises the impact of protein structure on nutrition and how various factors can alter protein structure, ultimately affecting nutrition.

## 2. Nutritional Requirements for Various Population Groups

Dietary protein, rich in essential amino acids, is crucial for body metabolism, growth, development, and health. Ensuring adequate protein supply at individual and population levels is vital for global food and nutrition security [12]. Also, due to variables such as age, sex, physiological changes, and lifestyle, various demographic groups have varying nutritional needs. According to the National Academy of Sciences, the recommendations for Dietary Reference Intakes (DRIs) are specific to the various stages of life. It should be noted that DRIs are for healthy individuals, not those chronically ill or at high risk due to age, genetics, or lifestyle factors such as smoking, alcohol intake, or strenuous exercise [13]. Below is a detailed overview of the nutritional requirements for various population groups, including adults (females and males), older adults, children, infants, and other special groups.

### 2.1. Adults (Females and Males)

According to Canêo and colleagues [14], the World Health Organization (WHO) defines an adult as someone older than 19 years, unless national law delimits an earlier age. Adult energy needs vary based on age, physical activity, and basal metabolic rate, with males needing more energy than females. The Recommended Dietary Allowance (RDA) for protein intake is 0.83 g per kilogram of body weight per day (g/kg bw/day) for both males and females. Whole-body protein mass maintenance relies on equal rates of protein synthesis and breakdown, resulting in a zero net balance. The first official recommendation for protein intake of 10 g/kg bw/day, published in 1936 by the League of Nations, was set based on observations from practice rather than relying on data from a strictly scientific approach. Several joint Food and Agriculture Organization (FAO)/World Health Organization (WHO) expert committees have faced challenges defining protein requirements. Over the years, various recommendations have been made. However, the 2007 recommendation defined the protein requirement as the lowest level of dietary protein intake that will balance the losses of nitrogen from the body and thus maintain the body protein mass in persons at an energy balance with modest physical activity levels.

Table 1 is a summary of major guidelines from external expert bodies, which, however, are not official recommendations. The discrepancy in protein recommendations is partly due to the lack of a definitive criterion for “adequate protein” and differences in outcome measurements [15].

### 2.2. Older Adults

According to Honda and colleagues [23], an “older adult” person is defined as ≥ 75 years of age, based on the prevalence of comorbidities and the activities of daily living of people of this age. As we age, our calorie needs decrease due to decreased muscle mass and physical activity, but nutrient density remains crucial. The changes in body composition and mobility decrease energy requirements, predisposing individuals to inadequate dietary intake and protein and micronutrient deficiencies. Adequate protein intake (1.0–1.2 g/kg bw/day) is essential to prevent muscle loss and maintain overall health. As seen in Table 2, this is a major guideline from external bodies, though it is not an official recommendation. The discrepancy in protein recommendations is partly due to the lack of a definitive criterion for “adequate protein”, as well as differences in outcome measurements [15].

### 2.3. Children

Hudson and colleagues [18] defined persons whose ages are <19 years old as “children”. Energy requirements are of paramount importance for their overall growth and development. Accurate estimation of children’s energy needs is crucial for healthy growth and development, considering age, gender, physical activity, and health status. Protein is a crucial macronutrient for children’s growth, tissue repair, and overall development, forming muscles, enzymes, hormones, and antibodies. Factors including gender, activity level, and body weight influence protein requirements in children. Protein intake is crucial for infants’ first year to support their rapid growth. Breast milk, with a low protein content of 1.2 g per 100 mL, is highly digestible and suitable for the infant’s immature digestive system [26]. As cited by Milani and colleagues [27], protein intake in the first year of life is, indeed, considered one of the main determinants of growth later in life. Thus, the protein intake of breast-fed infants has been used as a model for infant protein requirements, given that breast milk is typically the only source of protein before complementary foods are introduced [28].

Post-first year, protein needs decrease slightly, while adolescents experience growth spurts. Boys may require more protein than girls due to their higher muscle mass and growth rate [29]. Active children who engage in sports or physical activities may require additional protein to support muscle repair and growth [30] and ideal body weight may need to be considered when calculating protein requirements for overweight or obese children [31].

Also, an inadequate protein intake can lead to growth retardation in children, affecting both height and weight [16]. Proteins play a vital role in supporting the immune system, and a deficiency can negatively impact immune function [32,33]. According to Duerden and colleagues [34], adequate early protein intake is essential for optimal brain growth, although adverse effects of protein overfeeding cannot be excluded.

The Australian Government’s National Health and Medical Research Council [25] recommends protein intake for different age groups. Infants aged 0–6 months should consume 1.52 g/kg bw/day, 7–12 months’ intake is 1.2 g/kg bw/day, while children aged 1–3 years should consume 1.1 g. Children aged 4–8 years should consume 0.95 g, while those aged 9–13 should consume 0.85 g/kg bw/day. Adolescents aged 14–18 should consume 0.75 g/kg bw/day for females and 0.84 g/kg bw/day for males. Adults aged 19 and above should consume 0.84 g/kg bw/day.

### 2.4. Other Groups

Protein requirements for specific groups such as pregnant women, athletes, vegetarians and vegans, older adults, athletes, and bariatric surgery patients are peculiar to their needs. Pregnant women’s requirements are elevated to support foetal growth and maternal physiological changes. The Recommended Dietary Allowance (RDA) for protein during the first trimester of pregnancy is estimated at 46 g/day (0.8 g/kg bw/day), and 71 g/day (1.1 g/kg bw/day) during the second and third trimesters [35]. Although protein requirement data specific to pregnancy are lacking because of practical and ethical concerns, the Estimated Average Requirement (EAR) for protein in healthy pregnant women at early and late gestation was determined to be 1.22 g/kg bw/day (r^2^ = 0.60; 95% CI: 0.79, 1.66 g·kg^−1^·d^−1^) and 1.52 g/kg bw/day (r^2^ = 0.63; 95% CI: 1.28, 1.77 g·kg^−1^·d^−1^), respectively [36].

For athletes, the International Society of Sports Nutrition (ISSN) recommends protein intake ranging from 1.2 to 2.2 g/kg bw/day [37]; for vegetarians and vegans, the Academy of Nutrition and Dietetics (AND) suggests that well-balanced vegetarian and vegan diets can meet protein needs, but attention to protein-rich plant sources such as legumes, tofu, and tempeh is essential [38]. Older adults who engage in regular exercise may require higher protein intake to combat age-related muscle loss, and rapidly digestible proteins rich in branched-chain amino acids are important. Recent research by Holwerda and colleagues [39] suggests that older athletes benefit from a protein intake of around 1.2 to 1.6 g/kg bw/day to maintain muscle mass. For bariatric surgery patients, protein requirements need to increase to maintain lean body mass and promote healing. Recent recommendations from the American Society for Metabolic and Bariatric Surgery (ASMBS) advise bariatric surgery patients to consume at least 60–80 g of protein per day [40].

## 3. Nutritional Measures

Protein quality measures assess the nutritional value of proteins by evaluating their digestibility and the availability of essential amino acids. Various methods and indices have been developed to determine protein quality; these include the Protein Efficiency Ratio (PER), Biological Value (BV), Net Protein Utilization (NPU), Protein Digestibility-Corrected Amino Acid Score (PDCAAS), Digestible Indispensable Amino Acid Score (DIAAS), Protein Chemical Score, and Protein Quality Evaluation Methods by WHO and FAO. It should be noted that each metric has its advantages and disadvantages and that there is no one-size-fits-all solution. DIAAS is a more accurate protein quality assessment tool that considers amino acid content and digestibility, understanding the nutritional value of protein sources, and aiding in dietary recommendations [41] and food product development.

In this review, only the PDCAAS and DIAAS methods will be discussed because they are the most recent, most scientifically valid, and most commonly used today. The PDCAAS and the DIAAS of common food proteins are provided in Table 3.

### 3.1. Protein Digestibility-Corrected Amino Acid Score (PDCAAS)

The PDCAAS is a chemical score used to evaluate protein quality, derived from the ratio between the first limiting amino acid in a test protein and the corresponding amino acid in a reference amino acid pattern. It is truncated to 100% for scores exceeding 100%. The PDCAAS is simple and directly related to human protein requirements.

However, there are several limitations of PDCAAS [27]:

(a) The reference pattern is based on the minimum amino acid requirements for tissue growth and maintenance and does not necessarily reflect the optimum intake.
(b) Truncated PDCAAS of high-quality proteins do not provide any information about their power to compensate, as a supplement, for low levels of dietary essential amino acids in combinations with low-quality proteins.
(c) Faecal N digestibility does not consider the loss from the colon of indispensable amino acids not absorbed in the ileum.
(d) Anti-nutritional factors, such as lectins and trypsin inhibitors, found in several plant protein sources can cause heightened endogenous losses of amino acids. This issue is particularly relevant in animal feedstuffs but is also relevant in human foods, notably soy products.

### 3.2. Digestible Indispensable Amino Acid Score (DIAAS)

This method evaluates protein quality by measuring the true ileal digestibility of dietary indispensable amino acids. DIAAS is the ratio of dietary indispensable amino acids absorbed by the ileum to the amount of dietary indispensable amino acids present in a reference protein [46]. The DIAAS method has been proposed as a more accurate method than PDCAAS because it accounts for differences in amino acid digestibility between foods [47].

## 4. Protein Structure, Digestibility, and Bioavailability

A range of factors can affect protein digestibility. These are mainly the modification of amino acid sidechains, making them unavailable for nutrition and/or affecting the ability of digestive enzymes to bind, and the conformational structure of the protein, which can restrict digestive enzymes’ access to binding sites through steric hindrance [6,48].

Lysine is an important component of proteins, not only because it is an (often limiting) essential amino acid but also because it is an important part of the binding site for some proteolytic enzymes, notably trypsin. The loss of lysine through Maillard reactions occurs when there is a chemical reaction between carbonyl compounds, especially reducing sugars and compounds that possess a free amino group, such as amino acids and proteins. The ε-amino groups of the lysine residues of proteins are the main source of free amino groups in proteins, and the ease with which they take part in the reaction explains why the Maillard reaction is the most important route to nutritional damage of food proteins when heated in the presence of sugars. The Maillard reaction between sugars and amino acids occurs ubiquitously during food processing and storage and is critical in forming both desirable and undesirable pigments and flavours. The reduction of nutritional quality, such as a decrease in lysine by the Maillard reaction, is cause for concern [49]. Lysinoalanine (LAL) is an unnatural amino acid that can be formed during food processing when some proteins are exposed to alkaline conditions [50]. This is most important for caseins and their products, and for other phosphorylated proteins, as lysinoalanine is formed by the dephosphorylation of phosphoserine. Lysinoalanine has been shown to have toxic effects on some animals, including rats, chickens, and pigs; however, its most important effect is to make lysine, often a limiting amino acid, unavailable.

Cysteine can also participate in a Maillard-type reaction, forming products similar to Maillard reaction products and amino acid-sugar adducts. Cysteine can also undergo oxidation, forming disulfide bonds and thus cysteine. This can also lead to the rearrangement of existing disulfide bonds, altering structure and digestibility. Oxidation can occur during high heat, such as during baking or roasting. Oxidation can also result in the formation of cystine sulfoxide, rendering cysteine unavailable for nutrition. The loss of cysteine through the Maillard-type reaction can affect food composition, flavour, and nutritional value, as cysteine is a dietary essential amino acid [51].

Protein conformation also affects digestibility, and proteins having a tight globular form or a hydrophobic core are resistant to hydrolysis [46]. These structures occur naturally in important seed storage proteins, such as those found in legumes and cereals, where they are stored specifically as a nutritionally dense food source for the developing seed embryo and are mobilised by cleavage at a relatively small number of sites by specific plant proteases during germination [52]. Usually, such proteins are denatured by cooking, changing their structure and exposing potential enzyme cleavage sites. In addition, the low pH in the stomach helps with the denaturation of many proteins.

Processing can also change the structure and thus improve or reduce the digestibility, depending on the process and the protein [46]. The main effects of processing on protein structure are due to the effects of heat or shear, or a combination of these. Both are forms of energy input that disrupt and unfold the tertiary structure of proteins. This can expose hydrophobic regions that may clump together to form protein aggregates that are resistant to digestion. It can also lead to an exchange of disulfide bonds, linking protein molecules together in aggregates and preventing the protein from refolding correctly. Heating can also cause sidechain modification, as discussed above.

## 5. Future Food Proteins—Can They Fulfil Nutritional Needs?

It has been estimated that the world can currently produce enough protein to feed the entire population [53], and that protein insufficiency, in some parts, is due to issues with distribution, affordability, and political interference. By 2050, the growing global population will result in more than 2 billion additional food consumers [54]. Protein is a major component of the global food market, expanding at a compounded annual growth rate of 8.5% from 2023 to 2032 [55], driven by rising demand for high-protein foods. However, regarding nutritional value, the amount of protein found in food is not the only important parameter, but also its digestibility and amino acid profile, as noted above [56]. Currently, animal-derived proteins (e.g., meat, poultry, fish, eggs, and dairy foods) and plant-based proteins (e.g., beans, legumes, nuts, and seeds) are available globally. These proteins differ based on amino acid compositions and protein digestibility.

The livestock industry has experienced significant criticism because of the environmental impacts of Global Greenhouse Gas (Global GHG) emissions (notably methane from ruminants), water pollution, and cropland limitations [57]. Water use is also an important consideration in many areas. According to the report of Philipp and colleagues [58], the average water needed for the production of a tonne of meat is 15,500 m^3^ for cattle, 4800 m^3^ for pigs, 6100 m^3^ for sheep, and 4000 m^3^ for poultry, but a tonne of potatoes requires only approximately 250 m^3^ of water for plant growth. Moreover, several studies have reported that meat consumption has been associated with a variety of human diseases, including cardiovascular diseases [59], type 2 diabetes, certain cancers, and dementia [60]. These issues have changed consumers’ eating patterns, leading to an increase in vegan and flexitarian diets in developed countries. The source and production of future food proteins that meet a predicted increase in global protein demand has led to a range of initiatives to develop alternative foods.

Addressing these problems while producing more food for a growing population will require changes to current food systems, of which dietary choices have a significant role in contributing to environmental impacts, which could be lessened by consuming fewer overconsumed animal products and more plant-based foods while reducing excess energy intake and the amount of food wasted [61]. The creation of the Food-Based Dietary Guidelines (FBDG) was based on building consensus among various sectors and groups involved in public health to provide a general outline of the steps in the process, which can be adapted to the specific needs of a country or region. The goal is to have a set of guiding principles for food-based recommendations that lay out the overall policy agreed upon by various agencies and groups [62] and can be followed by practitioners and individuals making dietary choices. Examples of these guidelines are the New Zealand Ministry of Health’s Eating and Activity Guidelines for New Zealand Adults [63] and the Food-Based Dietary Guidelines in the WHO European Region [64].

### 5.1. Cultured Meat

Cultured meat, also known as in vitro, artificial, or lab-grown meat, aims to recreate the complex structure of animal muscles using only a few cells. A biopsy is taken from a living animal. The cells begin to divide after being cultivated in an adequate culture media that contains nutrients, hormones, and growth factors or foetal bovine serum (FBS) [65,66,67]. Some advocates claim that cultured meat is safer than regular meat since it is produced in an environment completely controlled by researchers or manufacturers [66]. The first cultured meat burger patty was prepared and tasted on social media in August 2013. This cultured meat product was based on 10,000 strips containing myotubes engineered in a hydrogel to aid cell-induced contraction and tissue alignment. Although they are recognised as significant ingredients, the scaffold materials, e.g., alginate, cellulose, or chitosan, affect the macronutrient content, particularly of protein, in the final product and can serve as a source of healthy dietary fibre [68]. However, because of its appearance, some ingredients, such as saffron, beetroot, and binders, need to be added to improve the flavour, colour, and texture of the mimic meat [69]. This cultured meat can be added to processed meat products to produce up to 25% of total protein [70]. However, most sensory panellists left the suggestion that the dry texture of a cultured meat burger patty can be tasted due to a lack of fat content. In addition, there are still some concerns about cost, as the serum used as a medium during the cell growing period is expensive and directly impacts the cost of cultured meat production [71]. The feasibility of commercialising cultured meat may improve in the future if a less expensive means of production can be developed.

A recent approach has used plant-based tissue engineering that mimics meat by deconstructing it into its fundamental components: muscle, fat, and connective tissue, and reconstructs them using a combination of plant proteins, fats, and polysaccharide materials. The muscle component is reassembled to mimic the anisotropic fibrous structure of beef, while the fat component is engineered through lipid encapsulation within a hydrocolloid matrix. Advanced manufacturing techniques, including additive manufacturing and robotics, are utilised for the precise spatial configuration and assembly of these components [72].

### 5.2. Plant-Based Proteins

The market for plant protein is predicted to grow from $4.6 billion in 2018 to $85 billion in 2030 [73]. Traditional plant-based protein can be obtained from many sources, such as legumes (soybean, chickpea, lupin) and seeds (hemp, chia, sesame). Soy protein has a well-balanced amino acid composition. It can be prepared in concentrate or isolate forms with >65% and 90% protein content, respectively [42]. However, soybean protein production does not meet the global demand as it has faced significant problems, e.g., the limitation of farmland, and it requires >90 days of growing before harvesting.

The search for new alternative plant-based proteins to replace the traditional ones has gained attention from researchers. *Wolffia globosa* is a duckweed that has long been consumed in Asian and African countries [74]. Duckweed contains approximately 0.3–0.4 g of protein per gram of dry weight [75]. In addition, it is rich in essential amino acids, including leucine, valine, and phenylalanine [76]. It can be produced and grown in a basin on non-arable land with the ability to tolerate harsh conditions. This duckweed is well-known as a fast-growing plant that requires a few weeks of growth to be harvestable. According to these advantages, it has a high potential to be developed as a future protein source for food ingredients, food supplements, and functional food and beverages [77].

Plant leaf protein has been considered as a potentially important source of protein. RuBisCo is the acronym for the enzyme ribulose bisphosphate carboxylase. This enzyme is ubiquitous in green plants and is responsible for converting carbon dioxide into sugars. It is considered the most abundant protein on earth and is the major protein constituent of all green leaves. The possibility of extracting this protein from leaves as a food source has been investigated several times. A general problem with its extraction from leaves is the robust cell wall structure, often coupled with plant phenolics that can destroy nutritional value; however, with novel technologies, these problems can potentially be overcome. Other possible protein sources include grass leaves and sugar beet leaves. Several groups have explored protein extraction from grasses. Sugar beet is one of the few plant commodities for which the leaves are still fresh at the time of harvesting. This is of crucial importance for the extraction of proteins from the leaves. The 2022 world sugar beet production was 261 million MT [78]. This would correspond to about 160 million MT of sugar beet leaves. The protein content of the leaves is estimated at 2.5%, so a potential of about 260,998,613 million MT of protein could be available.

Hemp (*Cannabis sativa* L.), a plant from the Cannabaceae family, is used widely for textiles and food [79]. Its protein contains high levels of glutamic acid and arginine, making it an interesting protein source for human nutrition and health [80]. With 12% arginine content, hemp protein exceeds other high-protein animal-derived or plant sources such as wheat, soy, egg white and whey [81]. The amino acid profile of hemp protein is comparable to high-quality protein sources such as egg white and soy proteins [81]. While the literature on the bioavailability of hemp protein is still scarce, the in vitro digestion of isolated hemp proteins showed greater digestibility than isolated soy proteins [82,83].

Most plant-based proteins are limited or deficient in one or more essential amino acids and are not well-balanced in essential and non-essential amino acids [5]; however, consuming varied plant proteins, especially legumes, can provide the same quality as animal proteins [84].

### 5.3. Single-Cell Protein (SCP)

Single-cell protein is the term for the microbial protein biomass or bioprotein produced by algae, bacteria, yeast, and fungi [85]. These single-cell proteins can be used as protein-rich supplements or ingredients for human food and animal feed. Single-cell protein has been commercially available for some time, with Quorn, a product from the filamentous fungus *Fusarium venenatum*, having been available for human consumption in the UK since 1985.

Depending on the species and growth conditions, microbial or microalgal mass contains high protein content ranging from 30 to 80%, along with the other two primary macronutrients, lipids, and carbohydrates [86]. The total protein contents and composition of single-cell protein vary with microbial sources, e.g., single-cell protein from fungi and yeast contain 50–55% protein content (based on dry weight) with a small amount of methionine and cysteine, whereas bacterial single-cell protein contains 60–80% protein (dry weight) with a high content of methionine and lysine [87]. The production of proteins can be sustained over a year, but the yield might fluctuate due to seasonal and climatic changes [88]. SCP has been recognised as an eco-friendly protein because it can be produced using agricultural waste and by-product streams [89]. Moreover, producing these proteins requires less farmland and a shorter harvesting period than livestock and plants. Therefore, the scaling-up of single-cell protein production as an alternative protein source has received significant attention from food manufacturers worldwide.

### 5.4. Insect Protein

Insects suitable for consumption offer a hopeful avenue for protein provision, capable of broadening dietary options, enhancing economic opportunities, bolstering food and nutrition stability, and exerting a lesser environmental impact compared to alternative protein sources [90]. Recently, a little more than 1850 species of insects have been consumed by around 1.9 billion people worldwide [91]. Edible insect protein preparations contain a high level of protein, about 40–70%, on a dry weight basis. Insect proteins also meet the WHO essential amino acid content requirement, (Table 4), which measures the quality of a protein source by comparing its amino acid profile with the human requirements [16]. Furthermore, insect proteins are more digestible (79–98%) than plant-based proteins but are only slightly less digestible than animal-based proteins such as beef and egg white (100%) [92]. In addition, cricket (*Gryllus bimaculatus*) protein meets the requirements for human adults, and its amino acid sequence shows a prevalence of hydrophobic amino acids (50–100%) such as valine and leucine in the peptide chains, accounting for its high antioxidant activity [93]. Furthermore, the black soldier fly (*Hermetia illucens*) is one of the edible insect species chosen as the most promising for the commercial production of proteins, with numerous beneficial advantages to humans and the environment. This insect has a high bioconversion efficiency to convert organic waste into high-value biomass, of which the composition may depend on the substrate. The larvae are rich in fat and protein with abundant essential amino acids including leucine, lysine, and valine, making it an excellent protein source for daily intake exceeding FAO recommendations [94,95]. Depending on the feed provided, the larvae can grow and be ready for harvesting within one month. Thus, these edible insects are a possible source of future protein that supports policies that encourage more sustainable and efficient food production and consumption.

### 5.5. Can Future Food Proteins Cause Food Allergies?

A food allergy is described as an adverse human health consequence caused by an immune reaction to specific dietary proteins. Food allergies are largely caused by large antigenic peptides arising from partial digestion of proteins in the stomach. These often have a high content of proline, which limits polypeptide chain flexibility, thus hindering access to digestive enzymes. These peptides can react with immunogenic cells in the small intestine regions known as Peyer’s patches, resulting in allergies or intolerances.

Most reported and confirmed food allergies are IgE-mediated because they cause the immune system to release Immunoglobulin E (IgE) antibodies [96]. In addition, the risk factors driving allergic reactions are primarily three factors that affect protein sensitisation and the development of protein allergy: the intrinsic and immediate environmental factors, exposure routes, and the allergen itself. Another individual vulnerability is determined by a genetic or epigenetic phenomenon known as the atopic phenotype [97]. Traditional plant-based foods are the most common cause of food allergy in humans. However, future proteins mentioned above could expose allergic and non-allergic consumers to new food allergens. As the reported number of allergic consumers has increased, new alternative plant-based foods may need an intensive clinical examination before launching those future proteins to the market [97,98].

## 6. Conclusions

The importance of considering both the quantity and quality of protein sources for a healthy diet has been highlighted by recent research. Alternative protein sources are emerging as potential solutions to meet the increasing demand for protein while reducing the environmental impact of traditional animal agriculture. Recent investigations have focused on the bioavailability of specific plant-based proteins and their potential to meet nutritional requirements. However, more research is needed to fully understand the nutritional quality of alternative protein sources and how that is affected by processing due to growing interest in their potential to meet protein requirements and support global food security.

## Figures and Tables

**Table 1:** Successive protein requirements and recommendations by international groups to ensure nitrogen balance in adults.

Age	Recommended Dietary Protein (g/kg/d)	Reference
Adults	0.8	[2]
Adults (men and women)	0.83	[16,17]
Children		
(<19 years)	0.85–1.2	[18]
(6 months–18 years)	0.82–1.31 (f) 0.85–1.31 (m)	[16,17]
(0–6 months)	1.14–1.77	[16,17]
Individuals with chronic illness/malnourished	1.2–2.0	[19]
Older adults	1.00–1.30	[20]
Athletes	1.3–1.7	[21]
Vegetarians	0.99	[22]

**Table 2:** Protein recommendations by expert bodies to maintain body mass and strength in healthy older adults.

Age	Study Design	Recommended Dietary Protein (g/kg/d)	Report	Reference
>65 years	Nitrogen balance studies	0.83	WHO/FAO/UNU	[16]
>65 years	Epidemiological studies clinical trials	1.0–1.2	The ESPEN Expert Group, 2014	[15]
>65 years	Meta-analyses of nitrogen balance studies	1.0	The Nutrition Societies of Germany, Austria, and Switzerland (D-A-CH)	[24]
>70 years (men, women)	Factorial method	1.07, 0.94	Nutrient Reference Values for Australia and New Zealand	[25]

**Table 3:** Digestible Indispensable Amino Acid Score (DIAAS) and Protein Digestibility Corrected Amino Acid Score (PDCAAS) of common protein sources.

Protein Sources	PDCAAS	DIAAS	References
**Animal proteins**			
Skimmed milk powder	100 *	123	[42]
Milk protein concentrate	100 *	141	[42]
Casein		117	[43]
Whey protein isolate	100 *	125	[42]
Whey		85	[43]
Whey protein concentrate	100 *	133	[42]
Pork		117	[43]
Beef		112	[6]
Chicken	100	108	[6,44]
Egg	100	101	[43,44]
Insect protein		75	[6]
**Plant proteins**			
Wheat	51	54	[42]
Oats	64	57	[43,44,45]
Oat protein concentrate	69	67	[42]
Soy		91	[43]
Soy flour	100 *	105	[42]
Soy protein isolate	100 *	98	[42]
Pea	64	70	[43]
Pea protein concentrate	84	73	[42]
Fava beans		55	[43]
Rapeseed		67	[43]
Lupin	40–80	68	[43,45]
Canola		72	[43]
Corn		36	[6]
Potato	100	100	[43,45]
Gelatine		2	[43]
Red kidney beans		51	[44]
Chickpeas		85	[44]
Split red lentils		50	[44]
Split yellow peas		73	[44]

Values with asterisks (*) are truncated values. Digestible Indispensable Amino Acid Score (DIAAS), Protein Digestibility Corrected Amino Acid Score (PDCAAS).

**Table 4:** The essential amino acid requirements for an adult, recommended jointly by the Food and Agriculture Organization (FAO) of the United Nations, World Health Organization (WHO) & United Nations University (UNU) [16].

Amino Acid	Requirement (mg/kg/day)
Isoleucine	20
Leucine	39
Lysine	30
Methionine + Cysteine	10.4
Phenylalanine + Tyrosine	25
Threonine	15
Tryptophan	4
Valine	26

## Data Availability

The original contributions presented in the study are included in the article, further inquiries can be directed to the corresponding author.
